# Case report and literature review: Horner syndrome subsequent to endoscopic thyroid surgery

**DOI:** 10.1186/s12893-020-01042-w

**Published:** 2021-01-13

**Authors:** Yu Min, Hang Chen, Xing Wang, Ying Huang, Guobing Yin

**Affiliations:** 1 Department of Breast and Thyroid Surgery, The Second Affiliated Hospital of Chongqing Medical University, No.74, Linjiang Rd, Yuzhong Dist, Chongqing, 404100 People’s Republic of China; 2 Department of Pathology, The Second Affiliated Hospital of Chongqing Medical University, No.74, Linjiang Rd, Yuzhong Dist, Chongqing, 404100 People’s Republic of China

**Keywords:** Horner syndrome, Endoscopic thyroid surgery, Thyroid, Thyroid cancer, Complication

## Abstract

**Background:**

Horner syndrome (HS), mainly characterized by symptoms including ptosis, miosis, and anhidrosis on the affected face, is a condition that is well documented but rarely reported as a postoperative complication of thyroidectomy, particularly in endoscopic thyroid surgery (ETS). We hereby report a case of HS due to ETS with a brief literature review on this topic.

**Case presentation:**

A 31-year-old female was admitted to our hospital with an unexpected physical examination finding of two thyroid nodules that were hypoechoic, had an irregular shape, and exhibited calcification. Subsequently, the results of a fine-needle aspiration (FNA) biopsy from the thyroid nodules and BRAF^V600E^ mutation further confirmed the malignancy of these nodules. Thus, total thyroidectomy combined with central lymph node dissection (CLND) by ETS via the bilateral axillo-breast approach was performed on this patient. Histology confirmed the diagnosis of papillary thyroid microcarcinoma (PTMC) concurrent with Hashimoto’s thyroiditis (HT). However, this patient developed HS with ptosis in her left eye on postoperative day 3. All symptoms gradually resolved before the 3-month follow-up.

**Conclusion:**

HS subsequent to ETS is a rare complication. Thus, standardized and appropriate operative procedures, as well as subtle manipulation, are essential in preventing and reducing the occurrence of HS. In addition, the early diagnosis and management of this rare complication are also important for a favorable outcome.

## Background

Horner syndrome (HS) was first formally described by Horner in 1869 [1], and the classic symptoms include partial ptosis (drooping of the upper eyelid), miosis (constricted pupil), and ipsilateral facial anhidrosis (loss of the ability to sweat normally from one side of the face). HS [2] mainly occurs due to impairment of the oculosympathetic pathway. Clinically, HS is usually observed in patients with large neck masses or those undergoing head and neck surgery. For instance, Kaelin et al. [3] reported the first case of HS subsequent to thyroidectomy in 1915. Since then, approximately 25 cases of HS associated with thyroidectomy have been reported in the published literature. In general, the incidence of thyroidectomy-related HS is low, as it is approximately 0.2% [4]. With outstanding postoperative neck cosmetic results, minimally invasive thyroid surgery, such as endoscopic thyroid surgery (ETS) and robotic-assisted endoscopic thyroidectomy (RAET), has been widely introduced to clinical practice in recent years [5]. However, postoperative complications, such as HS, were also observed with these novel surgical approaches [6, 7]. Therefore, standard surgical procedures and detailed postoperative follow-ups are needed to effectively avoid such surgical technique-related complications.

In the present study, we report a female patient who developed HS secondary to ETS. In addition, we also discuss the possible causes of this rare complication, its management and the follow-up results based on a comprehensive literature review and our own experiences.

## Case presentation

**Fig. 1:**
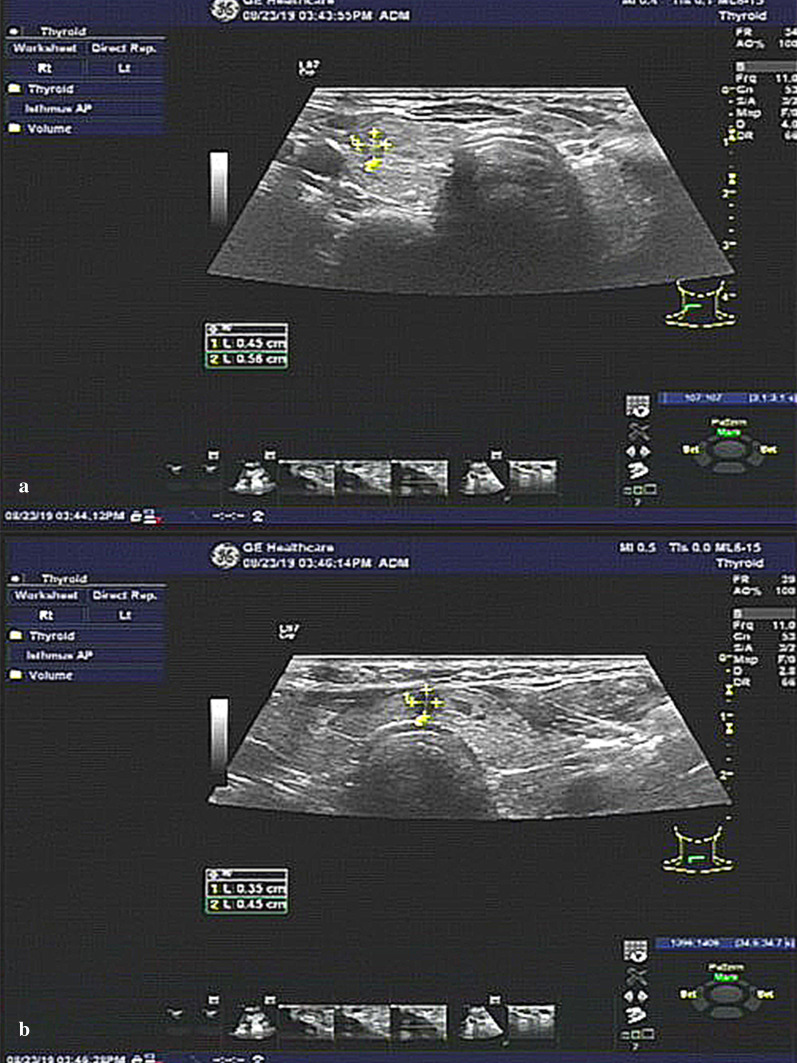
**a** Ultrasound imaging demonstrated a nodule (0.51 × 0.47 × 0.47 cm) that was located at the middle and upper part of the right thyroid gland; **b** Ultrasound imaging demonstrated a nodule (0.50 × 0.54 × 0.28 cm) that was located at the isthmus of the thyroid

A 31-year-old Chinese female was admitted to the Department of Breast and Thyroid Surgery with the unexpected discovery of two thyroid nodules (0.51 × 0.47 × 0.47 cm and 0.50 × 0.54 × 0.28 cm, respectively) during a routine physical examination 7 days prior (Fig. 1). The Thyroid Imaging Reporting and Data System (TI-RADS) scores of the nodules were classified as 4b and 4b, respectively. The patient was in a good general condition and did not have a history of chronic illness or smoking or drinking habits. Additionally, she did not complain about neck swelling or other discomforts when her medical history was collected. Only the serum levels of the thyroid peroxidase antibody (TPOAb, 127.6 IU/ml, reference 0.0–34 IU/ml) and thyroid globulin antibody (TgAb, 438.9 IU/ml, reference 0.0–115 IU/ml) were elevated according to the laboratory test results. After admission, a fine-needle aspiration (FNA) biopsy and BRAF^V600E^ gene test were performed. The results (atypical cells and BRAF^V600E^ mutation) revealed that these nodules were strongly suspected to be malignant.

**Fig. 2:**
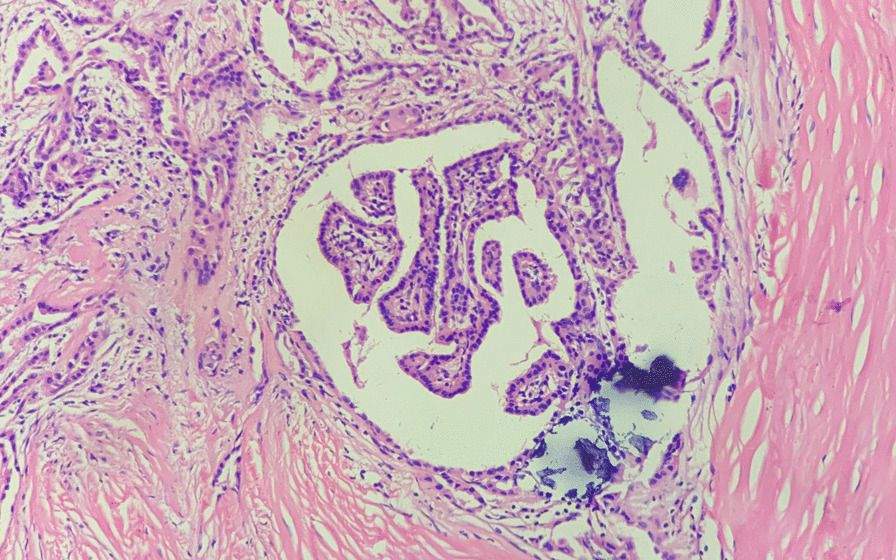
Microscopic image of PTMC from this patient, HE staining, × 20 magnification

**Fig. 3:**
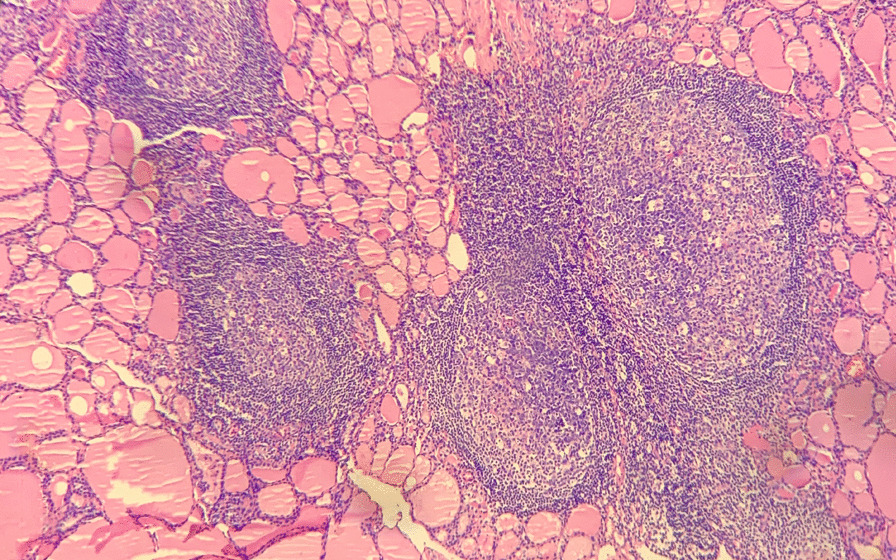
Microscopic image of HT (Hashimoto’s thyroiditis) from this patient, HE staining, × 10 magnification

Therefore, with for a goal of a satisfactory postoperative neck appearance, the patient underwent endoscopic thyroidectomy via the bilateral axillo-breast approach (BABA) on the 3rd day of hospitalization. Initially, the operational vision and manipulation spaces were established through two subcutaneous tunnels in the chest wall. Then, with the aid of endoscopy, a coagulation hook was used to separate the neck white line and anterior cervical muscles and further expose the whole thyroid gland. The two nodules were located in the superior part and the isthmus of the right thyroid gland, and they had hard and fixed surfaces and unclear boundaries. Thus, the right lobe and isthmus of the thyroid gland were simultaneously removed by an ultrasound knife, and then, all the samples were sent for intraoperative frozen section biopsies (FSBs). The results of the FSBs suggested papillary thyroid microcarcinoma (PTMC, Fig. 2) and Hashimoto’s thyroiditis (HT, Fig. 3). For this reason, the central lymph node (CLN) and residual thyroid tissue were resected. The postoperative pathological examination findings were consistent with the FSBs results and did not show lymph node metastasis (0/2).

**Fig. 4:**
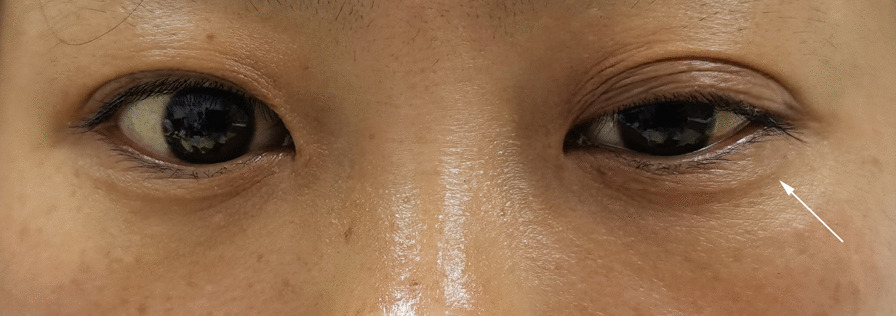
A 31-year-old female patients who underwent ETS, and suffered from ptosis (white arrow) on the 3rd day after the operation

However, on the 3rd postoperative day, the patient complained of upper eyelid drooping on the left side (Fig. 4). Thus, a comprehensive ocular examination was performed by a neurologist and ophthalmologist together. The left upper eyelid ptosis covered the corneal limbus for nearly 4 mm with an unequal pupil diameter (right: 4 mm, left: 2 mm). In addition, ipsilateral anhidrosis was also confirmed on the face. These symptoms led to the final diagnosis of HS. Hence, the patient received a detailed and reasonable explanation of this rare complication and was told that HS is a curable symptom. Then, she agreed to receive adjuvant neurotrophic therapy with mecobalamin (0.5 mg, oral administration, three times a day) and vitamin B1 (0.1 g, intramuscular injection, once a day) for 7 days before being discharged. No other complications, such as dyspnea, inflammation, or vocal cord palsy, were observed in this patient during the 12-day hospitalization period.

During the first 3-month follow-up, all symptoms of HS were significantly resolved without recurrence, and the patient did not report other symptoms, such as vision loss, photophobia, or cognitive dysfunction. In addition, she appreciated the timely diagnosis and treatment of HS and was fully satisfied with the aesthetic result of ETS. The timeline is described in Table 1.

**Table 1:** The timeline of the diagnosis and treatment process

Timeline	Diagnosis and treatment process
Dec 18th, 2019	Unexpected found two thyroid nodules
Dec 24th, 2019	Hospitalized in the department of Breast and Thyroid Surgery
Dec 25th, 2019	Fine-needle aspiration was performed
Dec 26th, 2019	Comprehensive department internal discussion
Dec 27th, 2019	Endoscopic thyroid surgery was performed
Dec 30th, 2019	The ptosis and anhidrosis were showed up on the left eye
Dec 31st, 2019	HS was diagnosed
Dec 31st, 2019	Neurotrophic support was immediately administered
Jan 05th, 2020	Discharged from the hospital
Mar 27th, 2020	The symptoms of HS were gradually resolved

## Discussion

### Epidemiology of HS

HS is a rare disease and occurs at a frequency of approximately 2.93 per 100,000 people, as reported in a recent nationwide population‑based cohort study [8]. In addition, the cumulative 10-year incidence of HS in the adult population was reported to be 2.95 per 100,000 adults. However, it is relatively lower in the pediatric population, with an incidence of 2.12 per 100,000 Korean children and 1.42 per 100,000 American children, respectively. Additionally, HS can occur at any age within any ethnic group [8, 9].

### Neuroanatomy and etiology of HS

Understanding the sympathetic innervation of the eye is pivotal to revealing the possible etiology of HS [10]. The oculosympathetic pathway contains three types of neurons. The first-order neurons are in the hypothalamus, with axons traveling through the brainstem and spinal cord that synapse in the lower cervical or upper thoracic spinal cord. Consequently, lesions stemming from the brainstem and cervical cord can present as first-order neuron HS. Then, second-order neurons originate from this spinal nucleus, travel through the upper chest cavity, and synapse in the superior cervical ganglion. For this reason, lesions in the upper chest cavity, such as pancreatic tumors, or iatrogenic interventions including but not limited to thyroidectomy and radical neck dissection can induce signs and symptoms of HS. In addition, third-order neurons originating in the superior cervical ganglion travel along the carotid artery system to reach the orbit and eye, which is close to the internal carotid artery. Therefore, pathologies in the neck, skull base, and orbit, as well as carotid diseases, can cause third-order HS [2].

Although the so-called typical risk factors for HS are well known, only approximately half of HS cases can be explained by a specific cause [8, 11]. Nonetheless, excluding HS cases with idiopathic and undetermined etiologies, the surgery procedure was the most frequent cause of adult HS, with a rate of 58% in the study by Han et al. and 24.7% in the study by Sabbagh et al. [8, 11]. This result was also observed clearly in the pediatric population [12]. Among these patients, cervical surgery plays the predominant role in the occurrence and development of HS [10], followed by direct invasion of the tumor, trauma, stroke, central venous catheter insertion, and carotid artery diseases. Notably, in one review article, thyroid pathologies were the most frequent causes of neck mass-related HS, accounting for approximately 1.3% of neck mass-related HS cases. [13]. Interestingly, however, when the main outcome measures were postoperative complications of neck surgery, especially thyroidectomy, the incidence of HS was significantly low. For instance, as two retrospective studies determined, HS only accounted for 0.2–0.3% of postoperative complications in patients who underwent thyroid surgery [4, 14]. With an increasing prevalence of thyroid malignancy around the world [15, 16], surgery remains an important initial choice for patients with thyroid carcinoma. Despite the significant development of minimally invasive surgical techniques for surgery, especially ETS with or without robotic assistance, some rare complications, such as HS, were still observed with these methods. To our knowledge, including the patient in our study, approximately twenty-five patients have developed HS due to thyroid surgery in recent years. In addition, only 4 cases among these patients were due to the ETS. The clinical characteristics of the cases are summarized in Table 2 [4, 6, 7, 14, 17–31]. Among these patients, the patients with HS most commonly underwent TT with lymph node dissection, especially with lateral lymph node dissection (LLND) [4, 6, 7, 14, 20, 23–26, 28–30]. In general, HS is not very difficult to diagnose in patients with a recent history of head or neck surgery and obvious symptoms during the postoperative hospital stay.

**Table 2:** Reported cases of Horner syndrome related to thyroid surgery

References	Case	Scopes of operation	Histopathology	Approach	Symptoms	Time to resolution
Zhang [17]	1	Nodule	Benign nodule	MWA	Myosis, ptosis	Incomplete resolved
Aslankurt [21]	1	ST	Nodular goiter	NM	Ptosis, anhidrosis	No improvement
Perréard [22]	1	ST	Benign nodule	NM	Miosis, ptosis	3 months
Cozzaglio [23]	1	TT	Toxic nodular goiter	NM	Ptosis, miosis	3 days
Italiano [24]	1	TT	Nodular goiter	Conventional	Myosis, ptosis, mild enophthalmos	2 months
Tan [7]	1	TT	FTC	ETS	NM	3 months
Seneviratne [25]	1	TT	Nodular goiter	NM	Ptosis, enophthalmos	12 months
Sandoval [26]	1	TT + CLND	ATC	Conventional	Ptosis, mild enophthalmos	NM
Meng [6]	2	TT + CLND	PTMC in 1 patient; PTC in 1 patient	ETS	a: miosis b: miosis, ptosis	11 and 1 months
Foma [27]	1	ET	Parapharyngeal ectopic goiter	Conventional	Miosis, ptosis, enophthalmos	12 months
Sapalidis [28]	1	TT + CLND + LLND	PTMC	NM	Miosis, ptosis	3 days
Mastronikolis [29]	1	TT + BLLND	MTC	Conventional	Miosis, ptosis	4 weeks
Lee [4]	5	2 with TT + CLND + LLND 3 with TT + BCLND + BLLND	Thyroid malignancy	Conventional	NM	NM
Hu [30]	1	Lobectomy + CLND + LLND	PTMC	ETS	Miosis, ptosis, enophthalmos	12 months
Kang [14]	1	NM	NM	RAET	NM	NM
Lee [31]	1	NM	NM	RAET	NM	NM
McCrory [19]	1	Lobectomy	Neuroma	NM	Ptosis miosis anhidrosis	Incomplete resolved
Punda [20]	1	TT + BCLND + BLLND	PTC	Conventional	Ptosis miosis anhidrosis	Incomplete resolved
Demiral [18]	1	TT	Nodular goiter	NM	Ptosis miosis	6 months
Present case	1	TT + CLND	PTMC	ETS	Miosis, ptosis	3 months

*TT* total thyroidectomy, *ST* subtotal thyroidectomy, *ET* ectopic thyroidectomy, *CLND* central lymph node dissection, *LLND* lateral lymph node dissection, *BCLND* bilateral central lymph node dissection, *BLLND* bilateral lateral lymph node dissection, *PTC* papillary thyroid carcinoma, *PTMC* papillary thyroid microcarcinoma, *FTC* follicular thyroid carcinoma, *ATC* anaplastic thyroid carcinoma, *MTC* medullary thyroid carcinoma, *NM* not mentioned, *MWA* microwave ablation, *ETS* endoscopic thyroid surgery, *RAET* robotic-assisted endoscopic thyroidectomy

### Clinical manifestations

As previously described, HS is a consequence of sympathetic disruption that can lead to a series of nerve dysfunction complications. The main symptoms of HS, including upper eyelid ptosis, miosis, and ipsilateral facial anhidrosis, are typical and can occur simultaneously or independently. In addition, the facial symptoms depend on the location of the lesion, and the severity depends on the degree of impairment. First, the superior tarsal muscle is responsible for keeping the upper eyelid in a raised position after the levator palpebrae superioris muscle raises it. Thus, upper eyelid ptosis can be accompanied by impairment of the superior tarsal muscle [2]. Second, miosis, another frequent symptom in HS, indicates a disruption in the sympathetic nervous system, which dilates the pupil. In contrast, the activation of the parasympathetic nervous system plays an independent role in making the pupil smaller. Consequently, the pupil in the affected eye is relatively smaller than that in the opposite eye due to the above reasons. Notably, the degree of anisocoria (unequal pupil size) is larger in darkness than in bright light because bright light normally causes both pupils to contract [2, 32]. Last, ipsilateral facial anhidrosis, another classic presentation, is not as frequent as the above signs. However, this symptom can be more apparent after strenuous exercise or a fever [2].

According to the present case, as well as other case reports, HS usually begins within a short time (from a few hours to 3 days) after surgery [6, 17, 21, 26, 28]. Myosis and ptosis are the most common self-reported symptoms and can be helpful for the early recognition and diagnosis of HS in postoperative management. Nonetheless, as HS is a nonlife-threatening complication, most of the symptoms are gradually relieved within a few months. However, it is also important to note that a minority of cases showed no improvement over a long-term follow-up period [17, 21].

### Diagnosis and differential diagnosis

First and foremost, the medical history of patients suspected of having HS needs to be evaluated carefully, as this step is regarded as the pivotal step in confirming a definite etiology, such as trauma, neck, or chest surgery. Moreover, except for significant clinical manifestations, several key pharmacologic tests can be significantly helpful to confirm HS, especially when the diagnosis is uncertain [33, 34]. Apraclonidine and cocaine eye drops are frequently used to confirm HS. Apraclonidine has an α-2 adrenergic agonist with weak α-1 activity. It has a negligible effect on pupil size. In contrast, in patients with HS, it significantly dilates the pupil because of the super- sensitivity of the iris dilator muscle from the upregulation of α-1 postsynaptic receptors. Compared with apraclonidine, cocaine can block the uptake of norepinephrine by the presynaptic membrane and further increase the amount of norepinephrine in the synaptic cleft, dilating the pupil and raising the eyelid. Therefore, the cocaine test relies on comparing the dilation response to cocaine eye drops in an affected eye versus the normal eye. A patient can be considered positive for HS if the affected eye does not dilate as well as the normal eye. In contrast, some causes may also be involved in miosis, including but not limited to third nerve palsy and optic neuritis. In addition, for ptosis, aging-related mechanical drooping in the eyelid is a frequent sign that needs to be distinguished from HS in elderly people.

### Prevention and management

**Fig. 5:**
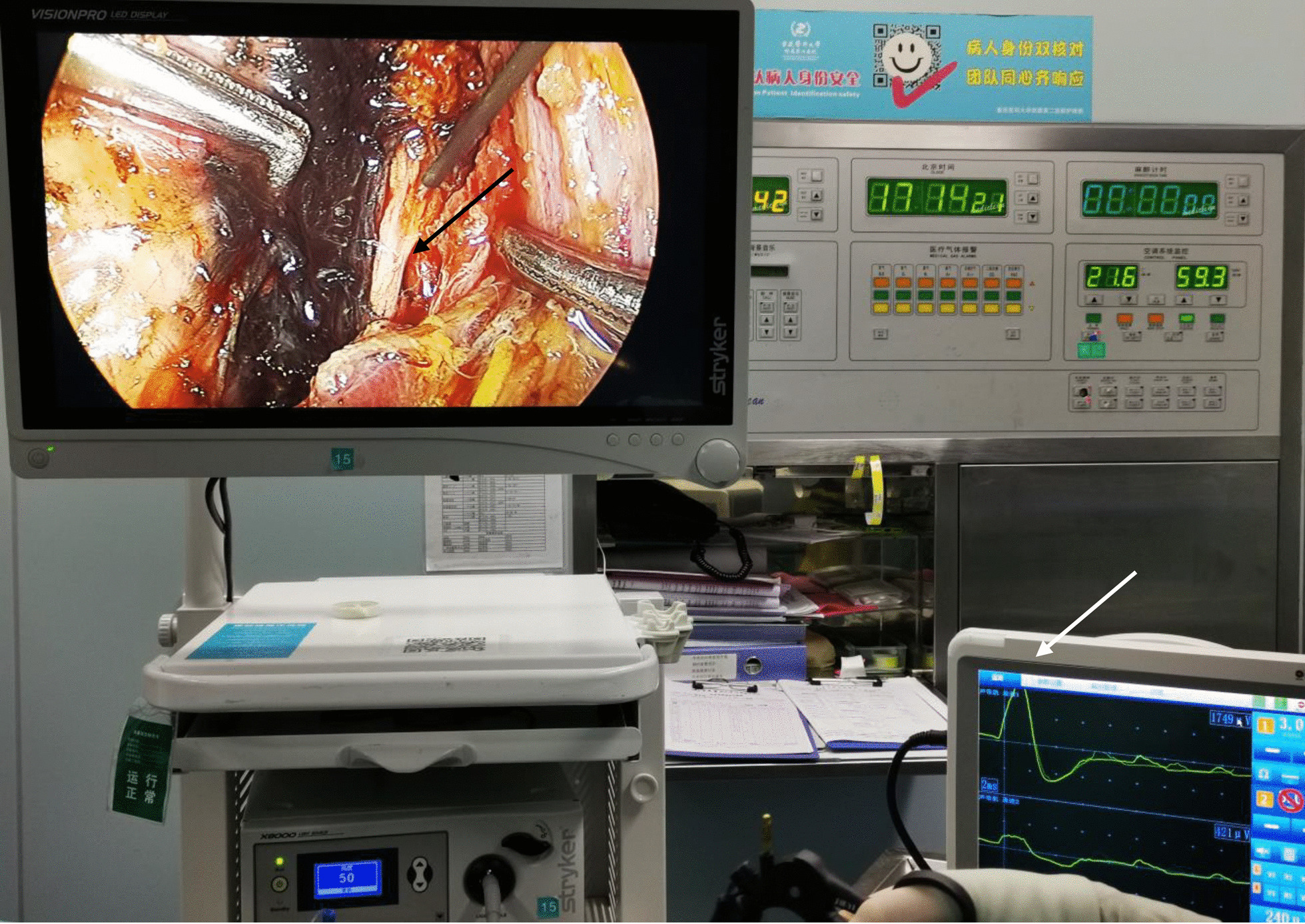
The intraoperative nerve monitoring equipment. The recurrent laryngeal nerve (black arrow) and voltage fluctuation after electrical stimulation (white arrow)

It is believed that perioperative management is crucially important for decreasing the risk of postoperative complications. Preoperatively, the surgeon needs to evaluate the nature, size, and location of lesions by conducting a comprehensive physical examination and imaging exams and then choose the appropriate type of operation. For instance, compared with ETS, conventional thyroid surgery can significantly improve the visualization of the surgical field and decrease the operation difficulty in patients with multiple central or lateral lymph node metastases. Intraoperatively, although ETS provides three-dimensional vision and a magnified view of the operative field, the difficulty of avoiding thermal accumulation is increased due to less tactile and strength feedback being provided for surgeons, which may result in thermal damage to surrounding tissues. As one network meta-analysis confirmed, the ultrasonic coagulation results are superior to other conventional techniques, except in decreasing the incidence of nerve injury [35]. For this reason, attention should be paid to maintaining a safe distance to the prevertebral fascia when using an ultrasonic knife to remove the thyroid lobe or performing lateral lymph node dissection. The target tissue or blood vessels should be carefully blunt separated from surrounding tissues so that we can safely perform thermal detaching. Notably, intraoperative nerve monitoring equipment (Fig. 5), one of the new types of equipment that was developed in recent years, can effectively help surgeons determine the location and degree of preservation of cervical nerves, such as the recurrent laryngeal nerve, superior laryngeal nerve, and cervical sympathetic nerve, during ETS. Postoperatively, HS is usually transient and insidious and does not result in the loss of visual function but shows cosmetic defects. Therefore, any self-reported complaints in patients, especially those associated with facial appearance, should be considered, as they can help in the diagnosis of HS in an early stage. Short-term neurotrophic therapy, such as vitamin B1 and vitamin B12 (mecobalamin), may help relieve these symptoms.

Generally, although HS is a rare complication due to thyroid surgery, especially ETS, surgeons must gain an understanding of the anatomic relationships of the cervical sympathetic and thyroid glands. In addition, standardized and appropriate operative procedures and subtle manipulation are essential in preventing and reducing the occurrence of HS. As shown in our case report, HS is not a life-threatening complication, and the majority of these cases gradually resolve within a few years. However, during this period, patients suffer from the symptoms of ptosis and miosis, which will have a negative impact on their appearance and mental health. Thus, the early diagnosis and management of this rare complication are also important for a favorable outcome.

## Data Availability

The datasets used and/or analyzed during the current study are available from the corresponding author on reasonable request.

## Abbreviations

HS: Horner syndrome
HT: Hashimoto’s thyroiditis
TI-RADS: Thyroid Imaging Reporting and Data System
FNA: Fine-needle aspiration
TPOAb: Thyroid peroxidase antibody
TgAb: Thyroid globulin antibody
FSB: Frozen section biopsy
TT: Total thyroidectomy
ST: Subtotal thyroidectomy
ET: Ectopic thyroidectomy
CLND: Central lymph node dissection
LLND: Lateral lymph node dissection
BCLND: Bilateral central lymph node dissection
BLLND: Bilateral lateral lymph node dissection
FTC: Follicular thyroid carcinoma
ATC: Anaplastic thyroid carcinoma
MTC: Medullary thyroid carcinoma
PTC: Papillary thyroid carcinoma
PTMC: Papillary thyroid microcarcinoma
MWA: Microwave ablation
ETS: Endoscopic thyroid surgery
RAET: Robotic-assisted endoscopic thyroidectomy
NM: Not mentioned
